# KRT6A Promotes Lung Cancer Cell Growth and Invasion Through MYC-Regulated Pentose Phosphate Pathway

**DOI:** 10.3389/fcell.2021.694071

**Published:** 2021-06-21

**Authors:** Di Che, Mingshuo Wang, Juan Sun, Bo Li, Tao Xu, Yuxiong Lu, Haiyan Pan, Zhaoliang Lu, Xiaoqiong Gu

**Affiliations:** ^1^Clinical Biological Resource Bank, Guangzhou Institute of Pediatrics, Guangzhou Women and Children’s Hospital, Zhongshan School of Medicine, Sun Yat-sen University, Guangzhou, China; ^2^Zhongshan School of Medicine, Sun Yat-sen University, Guangzhou, China; ^3^The School of Biomedical and Pharmaceutical Sciences, Guangdong University of Technology, Guangzhou, China; ^4^Key Laboratory of Diagnosis and Treatment of Severe Hepato-Pancreatic Diseases of Zhejiang Province, The First Affiliated Hospital of Wenzhou Medical University, Wenzhou, China

**Keywords:** NSCLC, LSD1, G6PD, MYC, KRT6A

## Abstract

Keratin 6A (KRT6A) belongs to the keratin protein family which is a critical component of cytoskeleton in mammalian cells. Although KRT6A upregulation in non-small cell lung cancer (NSCLC) has been reported, the regulatory mechanism and functional role of KRT6A in NSCLC development have been less well investigated. In this study, KRT6A was confirmed to be highly expressed in NSCLC tissue samples, and its high expression correlated with poor patient prognosis. Furthermore, overexpression of KRT6A promotes NSCLC cell proliferation and invasion. Mechanistically, KRT6A overexpression is sufficient to upregulate glucose-6-phosphate dehydrogenase (G6PD) levels and increase the pentose phosphate pathway flux, an essential metabolic pathway to support cancer cell growth and invasion. In addition, we discovered that lysine-specific demethylase 1A (LSD1) functions upstream to promote KRT6A gene expression. We also found that the MYC family members c-MYC/MYCN are involved in KRT6A-induced G6PD upregulation. Therefore, this study reveals an underappreciated mechanism that KRT6A acts downstream of LSD1 and functions as a pivotal driver for NSCLC progression by upregulating G6PD through the MYC signaling pathway. Together, KRT6A and LSD1 may serve as potential prognostic indictors and therapeutic targets for NSCLC.

## Introduction

Lung cancer is the most common cause of cancer related death in the world and China, especially among males (Torre et al., 2015; Feng et al., 2019). There are two main histological subtypes of lung cancer, known as small cell lung cancer (SCLC) and non-SCLC (NSCLC). Approximately 85% of lung cancer cases are attributed to NSCLC, and the most common subtypes of NSCLC are lung squamous cell carcinoma (LUSC) and lung adenocarcinoma (LUAD) (Molina et al., 2008). In the past few decades, the therapeutic treatments of NSCLC have made significant progress. However, the overall cure and survival rate of NSCLC remain low (Herbst et al., 2018). Studies have shown that the 5-year survival rate for NSCLC from stage I to stage IIIA was about 14–49%, whereas the survival rate for stage IIIB/IV was less than 5% (Ko et al., 2018). The high mortality of lung cancer patients is primarily due to the fact that more than half of NSCLC patients already developed metastasis at diagnosis (Herbst et al., 2008; Scrima et al., 2017). Therefore, exploring the molecular mechanisms of NSCLC invasion and metastasis will help improve the therapeutic response and survival of NSCLC patients.

The keratin 6A (KRT6A) is a type II keratin protein involved in the epidermalization of squamous epithelium (Fujii et al., 2002; Chang et al., 2011). Recently, studies have found that KRT6A plays an important role in cell migration, particularly keratinocyte migration (Wang et al., 2018). Silencing KRT6A expression can inhibit cell invasion and metastasis of nasopharyngeal carcinoma (Chen and Shan, 2019). More importantly, high KRT6A levels in lung adenocarcinoma is associated with an unfavorable patient prognosis (Xiao et al., 2017), and KRT6A promotes the growth and metastasis of lung adenocarcinoma through inducing the epithelial-mesenchymal transition (Yang et al., 2020). Nevertheless, the functional mechanism of KRT6A in NSCLC warrants further investigation. Our study demonstrated the relationship between high KRT6A expression and pathological progression of NSCLC, and dissected the molecular mechanism underlying KRT6A regulated invasion of lung cancer cells. These results may assist to better understand the functional roles of KRT6A in NSCLC growth and metastasis, paving the way for developing new therapeutics against this common malignancy.

## Materials and Methods

### Cell Lines and Clinical Tissue Sample

Two NSCLC cell lines (NCI-H1299 and A549) were purchased from Wuhan Procell Life Science and Technology Co. A549 cells were cultured in a Dulbecco Modified Eagle Medium (DMEM) with 10% fetal bovine serum (FBS) which purchased from ExCell Bio, China Co. H1299 cells were cultured in a RPMI 1640 medium with 10% FBS at 37°C and 5% CO_2_. Tissue specimens (*n* = 30) obtained from patients diagnosed as stage I–IV NSCLC who underwent surgery at The First Affiliated Hospital of Wenzhou Medical University were used for immunohistochemical staining. This study was approved by the Ethics Committee of The First Affiliated Hospital of Wenzhou Medical University.

### Reagents and Antibodies

Antibody against HA-Tag (1:1,000 times dilution) (Cat. 3724), β-actin (1:1,000 times dilution) (Cat. 3700), Flag-tag (1:1,000 times dilution) (Cat. 66008), KRT6A (1:1,000 times dilution) (Cat. ab93279) and E-cadherin (1:1,000 times dilution) (Cat. ab40772). Goat Anti-Mouse IgG H&L (HRP) (Cat. ab150113) and Goat Anti-Rabbit IgG H&L (HRP) (Cat. ab6721). LSD1 inhibitors ORY-1001 (Cat. S7795), SP2509 (Cat. S7680), GSK2879552 (Cat. S7796) were purchased from Selleck. shKRT6A was purchased from Shanghai Genesci Medical Technology Co., Ltd. G6PD promoter vector was a gift from Dr. Shuai Zhang (Tianjin University of Traditional Chinese Medicine, Tianjin, China). KRT6A promoter vector was obtained from Shandong Vigenebio Technology Co., Ltd.

### Western Blot Analysis

Western Blot Analysis was performed using standard techniques: H1299 and A549 cells were harvested with RIPA lysis buffer (Thermo Fisher) on ice for 30 min and then centrifuged at 12,000 rpm for 15 min at 4°C. Total protein was quantified by the bicinchoninic acid assay (Thermo Fisher Scientific). Protein lysates were separated by 12% SDS-PAGE and transferred to PVDF membranes (Millipore). The membranes were blocked with 5% non-fat milk for 1 h, after three times TBST washes then incubated overnight at 4°C with primary antibodies. The membranes were washed and incubated 2 h at room temperature with secondary anti-IgG antibodies. Signals were detected using Luminol substrate solution.

### Quantitative Real-Time PCR Analysis

H1299 and A549 cells were harvested for total RNA extraction with TRIzol reagent (Invitrogen). Purified RNA was converted to cDNA using PrimenScript^TM^ RT reagent Kit (Takara) according to the manufacturer’s protocol. qRT-PCR was performed using SYBR Green (Biotool) on a Bio-rad CFX96 Real-time PCR System. All gene expression levels were normalized using housekeeping gene, β-actin and the relative fold change were calculated using the 2^–△^^△^^*Ct*^ method. KRT6A, G6PD, ACTIN, c-MYC, and MYCN primers for RT-qPCR are provided in Table 1.

**TABLE 1:** Primer sequences for RT-qPCR.

**Gene**	**Primer sequence**
Actin	F:5′-CATCGAGAAATTGAGACGGTG-3′ R: 5′-CCTTGGAAGATGGTCTTGAT-3′
G6PD	F: 5′-TGAGTCAGACAGGCTGGAAC-3′ R: 5′-CACGGAAAAGAGAGGAGATG-3′
KRT6A	F: 5′-AATCGATCCCACCATCCAGC-3′ R: 5′-CTCCAGGTTCTGCCTCACAG-3′
c-MYC	F: 5′-CGTCCTCGGATTCTCTGCTC-3′ R: 5′-GCTGCGTAGTTGTGCTGATG-3′
MYCN	F: 5′-ATGACTTCTACTTCGGCGGC-3′ R: 5′-CCACAGCTCGTTCTCAAGCA-3′

### Luciferase Reporter Assay

Luciferase reporter assay was according to the manufacturer’s instructions of Dual-Luciferase Reporter Assay System (Promega). When H1299 cells were cultured to 70% confluence, they were seeded in 6-well plates and co-transfected with c-MYC/MYCN/LSD1 inhibitors with pGL3-G6PD-3’UTR, pGL3-KRT6A-promoter and Renilla luciferase plasmids using Lipo3000 (Sigma-Aldrich). Cells were lysed and each hundred microliters of protein extracts were analyzed in a luminometer for luciferase activity 48 h after transfection.

### EdU Analysis of DNA Synthesis

EdU assay was carried out to determine DNA synthesis level as previously described (Shan et al., 2019). H1299 cells transiently expressed KRT6A were incubated with EdU for 2 h. After fixation and permeabilization, the incorporated EdU was incubated with 1 × Apollo (Ribobio) staining reaction solution for 30 min at room temperature (RT), visualized by means of click reaction. The nuclear DNA was stained with 1 × Hoechst33342 for 60 min at RT. The images were obtained by Leica inverted fluorescence microscope. The data were analyzed by image analysis software.

### Cell Proliferation Assay

For cell proliferation assays, cells were seeded at the same number, 10 × 10 (Herbst et al., 2018) cells in six-well plates under the same conditions. Cell growth was determined by counting cell numbers at 0, 1, 2, 3, and 4 days after seeding.

### Transwell Assay

Transwell assay was carried out using transwell chambers (Corning) for detecting the invasiveness of H1299 cells and performed using 24 well transwell plates with polycarbonate membrane and 4.0 μM pores (Corning). After 24 h of LSD1 inhibitors treatment or 48 h of transfection, H1299 cells in serum-free RPMI 1640 medium were seeded in chambers with solidified matrigel (4.0 μg/μL, 60 μL) in advance for 3 h at RT. RPMI 1640 medium with 10% FBS was added to the lower chambers. H1299 cells in the upper chambers were at a density of 500 cells/per well. After 24 h, the medium from the lower chamber was removed and the upper chamber cells were wiped, then the migrated cells were fixed in 4% paraformaldehyde and stained with crystal violent for 30 min at RT. Migrating cell numbers were observed in four random fields under an inverted phase microscope (Leica) and cell numbers were enumerated using Image J.

### Lentivirus Production and Generation of Stable Cell Lines

To produce lentivirus, psPAX2, pMD2.G and lentiviral vector were packaged with Lipofectamine2000 and transfected into HEK293T cells in 10 cm dishes according to a standard packaging system. The virus medium was collected and filtered two times at 24 and 48 h after transfection. To establish stable cell lines, cells were infected with virus-containing medium (filtered virus medium and fresh medium 1:1, and added 5 μg/mL polybrene). To screen the positively infected cells, 5 μg/mL puromycin was added 72 h after infection and selected for 7 days.

The target sequence of shKRT6A is: 1#: 5′-GCTCTCAAACT CTCTAACTTA-3′, 2#: 5′-TCGCTGTTTGCAATTGCTAAA-3′, 3#: 5′-CTCCAGCAGGAAGAGCTATAA-3′.

### Bioinformatic Analysis

The public data set GSE19804 and the Gene Expression Profiling Interactive Analysis (GEPIA)^1^ were used for bioinformatics analysis. Overall survival analysis was performed by Kaplan-Meier Plotter^2^.

### Statistical Analysis

All data were representative of three independent experiments and presented as the mean ± standard error. Statistical analyses were calculated from Student’s *t*-test. *P* < 0.05 was considered to indicate a statistically significant difference.

## Results

### KRT6A Expression Is Altered in Lung Cancer

**FIGURE 1:**
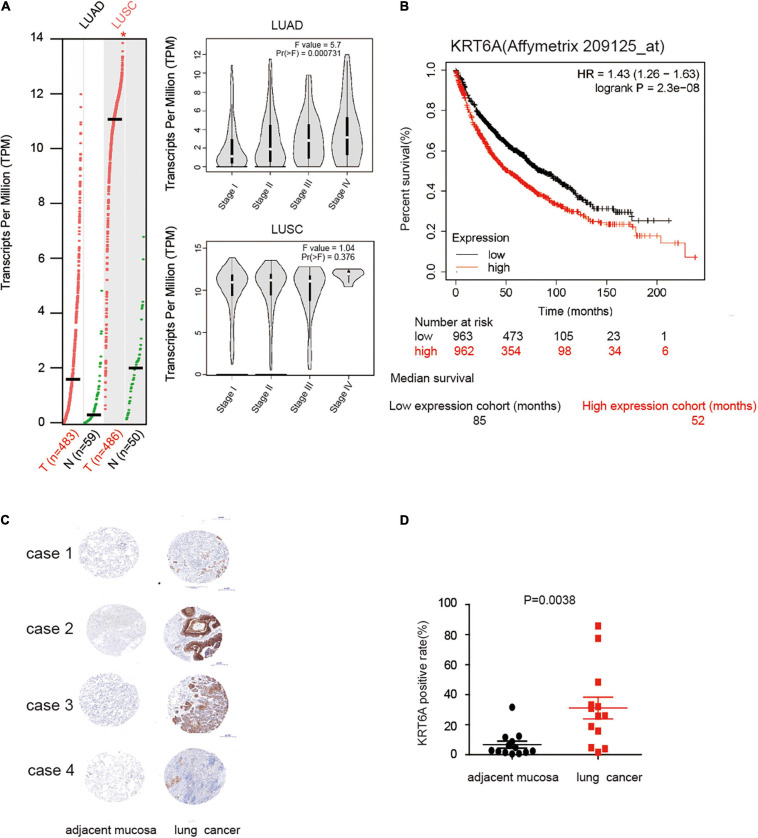
KRT6A is upregulated in NSCLC and associated with patient prognosis. **(A)** The levels of KRT6A in 483 lung adenocarcinoma and 486 lung squamous carcinoma tissues, and its expression differed according to TNM stages as recorded in (http://gepia.cancer-pku.cn/). **(B)** Kaplan-Meiers survival analysis of KRT6A expression in lung cancer patients with low and high tumor expression of KRT6A, calculated from (http://kmplot.com/analysis/). The cutoff lines to divide into high and low group was median value. **(C)** IHC analysis of KRT6A in NSCLC tissues (×400). Note low staining of KRT6A in normal tissues and high staining of KRT6A in NSCLC tissues. **(D)** Quantification of IHC data revealed significantly increased KRT6A expression in NSCLC specimens compared with the adjacent tissues.

To explore the role of KRT6A in lung cancer, we used expression data for KRT6A in lung cancer from The Cancer Genome Atlas (TCGA) database. KRT6A expression was significantly higher in lung adenocarcinoma (LUAD) and lung squamous cell carcinoma (LUSC) than normal tissues. Further analysis identified KRT6A expression also differed according to TNM stage. In LUAD, KRT6A were upregulated with increasing TNM stages, whereas KRT6A expression was significantly increased in advanced LUSC tumors (Figure 1A). Kaplan-Meier analysis shows that high levels of KRT6A expression was associated with poor patient prognosis, based on calculations from http://kmplot.com/analysis/. The median survival of patients with low KRT6A expression was 85 months and high expression was 52 months (Figure 1B). Furthermore, immunohistochemical staining results showed significantly increased levels of KRT6A protein in tumor tissues than normal adjacent tissues (Figures 1C,D; *p* < 0.05). In summary, these data suggested that KRT6A expression is highly correlated with poor prognostic factors in NSCLC.

### The Relationship Between KRT6A and Clinicopathological Characteristics in Patients With NSCLC

The clinicopathological characteristics of NSCLC patients are presented in Table 2, IHC analysis confirmed that high expression of KRT6A was associated with pathological stage (*p* = 0.001). Furthermore, Univariate and multivariate Cox regression analysis was performed in NSCLC patients. We found that patients with high KRT6A expression showed significantly high risk of death (univariate cox regression analysis: hazard ratio = 1.491, *p* = 0.016) (multivariate Cox regression analysis: hazard ratio = 1.854, *p* = 5.736e-05; Table 3). These results indicated that KRT6A could be used as a risk factor to predict the clinical outcome of NSCLC patients.

**TABLE 2:** Association between KRT6A levels and clinicopathological parameters in NSCLC patients.

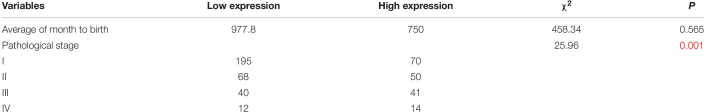

*Statistically significant values are shown in red (P < 0.05)*

**TABLE 3:** Cox regression analysis of risk factors for cancer related death in NSCLC patients.

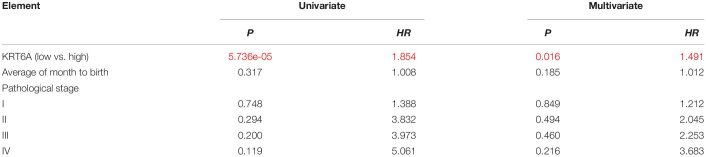

*Statistically significant values are shown in red (P < 0.05)*

### KRT6A Promotes Cell Proliferation and Invasion of NSCLC Cells

**FIGURE 2:**
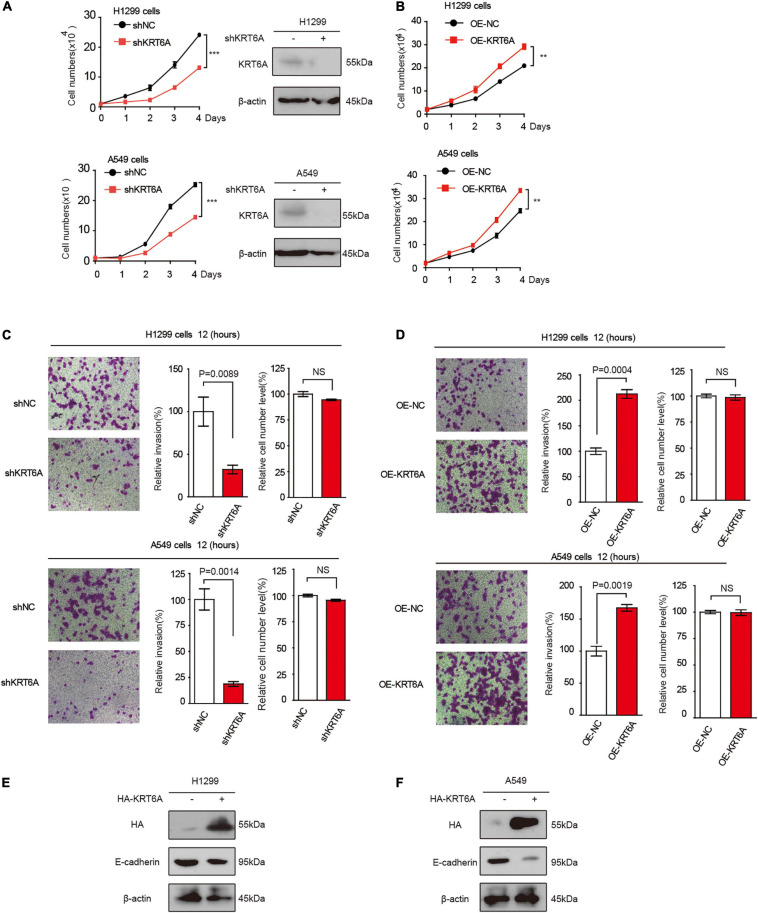
KRT6A promotes lung cancer cell proliferation and invasion. **(A)** Cell proliferation rates of human lung cancer H1299 and A549 cells with stable knockdown of KRT6A. **(B)** Cell proliferation rates of human lung cancer H1299 and A549 cells with stable expression of KRT6A. **(C)** Transwell assay of cell invasion was performed in human lung cancer H1299 and A549 cells with stable knockdown of KRT6A. **(D)** Transwell assay of cell invasion was performed in human lung cancer H1299 and A549 cells with stable expression of KRT6A. **(E,F)** Overexpression of KRT6A significantly decreases E-cadherin levels in H1299 and A549 cells (**0.001 < *p* < 0.01; ***p < 0.001).

To address the functional consequence of KRT6A upregulation in lung cancer, we established H1299 and A549 cells with KRT6A knockdown or overexpression. Our results show that depleting KRT6A inhibited cell proliferation of lung cancer cells (Figure 2A), and KRT6A overexpression promoted cell proliferation (Figure 2B). Furthermore, overexpression of KRT6A significantly promoted cell invasion (>twofold promotion, *p* < 0.01) (Figures 2C,D). Consistent with this result, we found that the expression of E-cadherin was decreased by transient overexpression of KRT6A (Figures 2E,F). These data indicate that KRT6A promotes lung cancer cell proliferation and invasion.

### LSD1 Promotes KRT6A Expression

**FIGURE 3:**
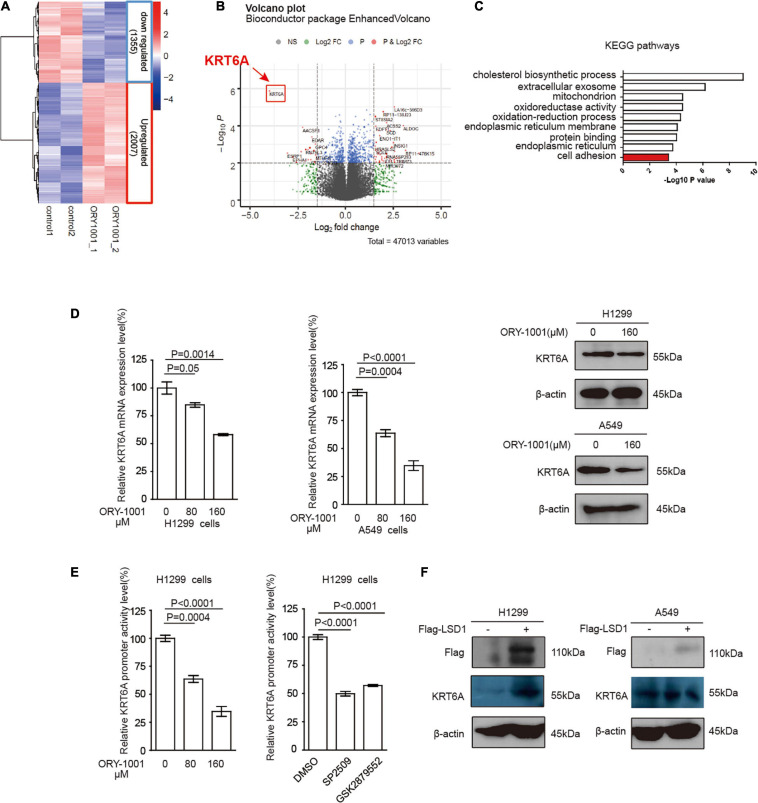
LSD1 is involved in regulating KRT6A expression. **(A,B)** The transcripts in H1299 cells was determined by RNA-sequencing, showing that KRT6A was significantly reduced in H1299 cells treated with a LSD1 inhibitor ORY-1001. **(C)** KEGG pathways analysis of the RNA sequencing results revealed that 10 significantly altered pathways were identified and the cell adhesion pathway was highlighted in red. **(D)** The mRNA and protein expression of KRT6A after ORY-1001 treatments were significantly lower compared to control in H1299 and A549 cells. **(E)** The LSD1 inhibitors (ORY-1001, SP2509, and GSK2879552) led to significantly reduced KRT6A promoter activity. **(F)** Overexpression of LSD1 promotes KRT6A protein expression.

LSD1 is an amine oxidase histone demethylase, and ORY-1001 is a potent and selective covalent inhibitor of LSD1 that suppresses the proliferation of acute leukemia cells (Fiskus et al., 2014). In this study, we investigated ORY-1001 treated and control H1299 cells with transcriptomic analysis via RNA-seq. After correction for multi-testing by controlling the false discovery rate at 0.05, we noticed that 2,007 genes were down-regulated, and 1,355 genes were up-regulated with ORY-1001 treatments (Figure 3A), with KRT6A being most significantly attenuated (Figure 3B). Pathway enrichment analysis revealed different pathways altered in ORY-1001 treated cells, including the cell adhesion pathway (Figure 3C). Quantitative real time-PCR and western blot analyses validated the suppression of KRT6A expression by multiple LSD1 inhibitors (ORY-1001, SP2509, and GSK879552) (Figure 3D). We also examined KRT6A promoter activity using a luciferase reporter, and found that LSD1 inhibitors significantly repressed KRT6A promoter activity (Figure 3E). Moreover, overexpression LSD1 could promote KRT6A protein expression in H1299 and A549 cells (Figure 3F).

### ORY-1001 Inhibits the Invasion of NSCLC Cells

**FIGURE 4:**
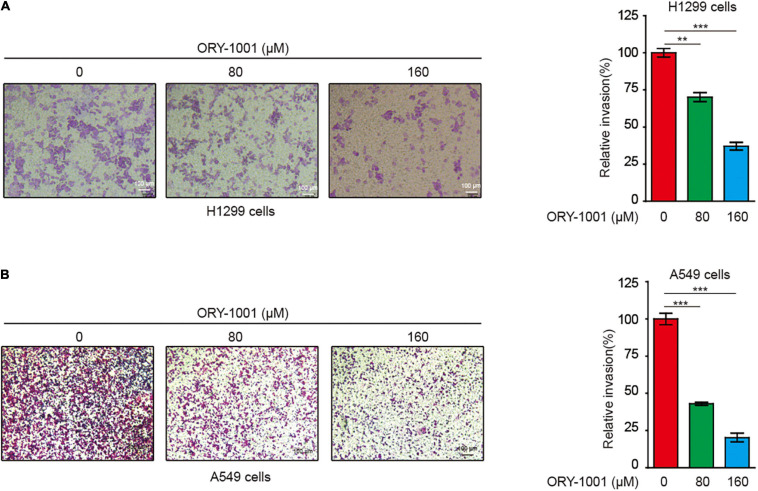
ORY-1001 inhibits NSCLC cell invasion. **(A)** Transwell assay of cell invasion was performed in human lung cancer H1299 cells treated with or without ORY-1001. **(B)** Transwell assay of cell invasion was performed in human lung cancer A549 cells treated with or without ORY-1001 (**0.001 < *p* < 0.01; ***p < 0.001).

Next, we conducted the transwell invasion assay to detect whether the LSD1 inhibitor could inhibit the invasion capability of lung cancer cells. As expected, ORY-1001 significantly inhibited the invasion of lung adenocarcinoma H1299 and A549 cells (Figures 4A,B).

### KRT6A Promotes G6PD Expression

**FIGURE 5:**
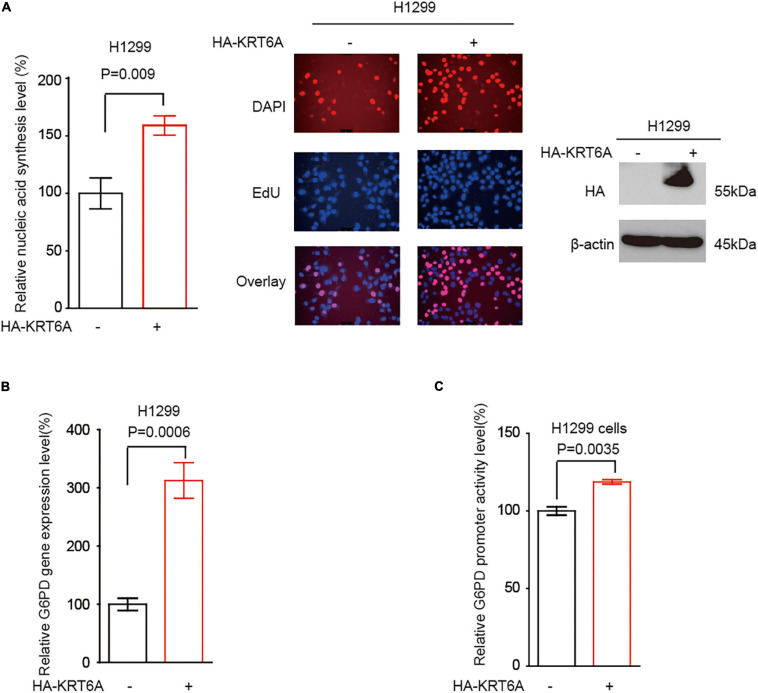
KRT6A promotes G6PD expression. **(A)** DNA synthesis was quantified in H1299 cells overexpressing KRT6A or an empty vector. **(B)** Determination of G6PD expression levels by Real-time PCR in KRT6A overexpressing and control H1299 cells. **(C)** Determination of G6PD promoter activity in KRT6A overexpressing and control H1299 cells.

It has become noted that the pentose phosphate pathway (PPP) plays a critical role in cancer cell invasion, partially through upregulating DNA synthesis (Shan et al., 2019). However, the detailed signaling event regulating PPP to promote invasion is far from clear. G6PD is the rate-limiting enzyme of PPP flux, which may facilitate cancer cell invasion. Accordingly, we speculated that KRT6A may regulate G6PD expression and PPP flux to stimulate invasion. Indeed, overexpression of KRT6A could enhance DNA synthesis (Figure 5A), increase G6PD transcript level (Figure 5B), and strengthen G6PD promoter activity (Figure 5C).

### KRT6A Promotes G6PD Expression Through Upregulating c-MYC/MYCN

**FIGURE 6:**
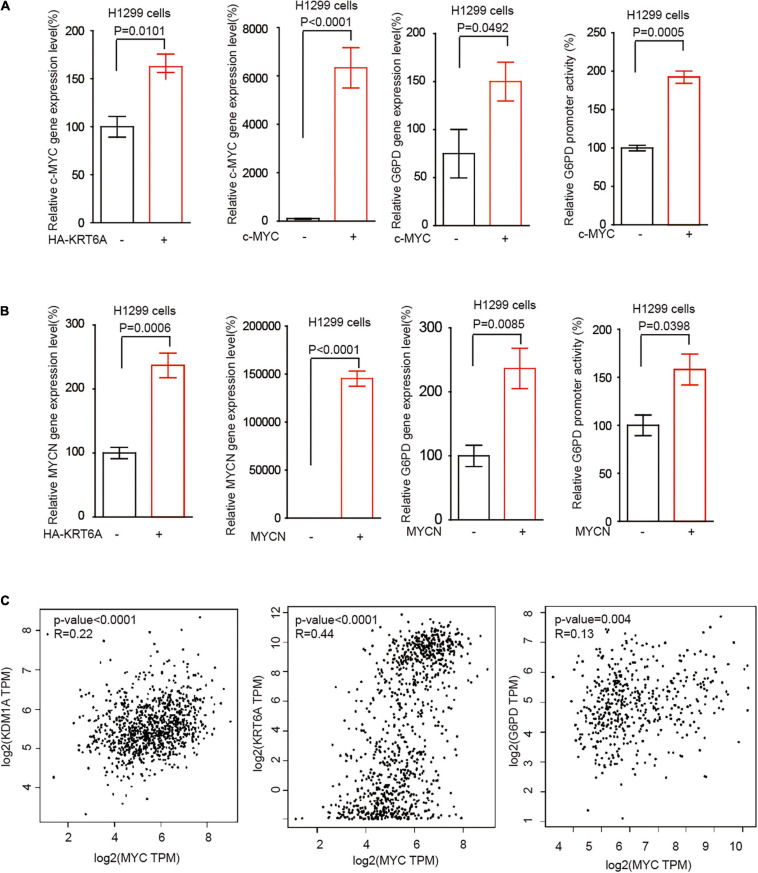
KRT6A promotes G6PD expression through upregulation of c-MYC and MYCN. **(A)** Determination of c-MYC mRNA expression levels by Real-time PCR (left) in KRT6A overexpressing and control H1299 cells. The G6PD mRNA expression level and G6PD promoter activity (right) in c-MYC overexpressing and control H1299 cells. **(B)** The MYCN mRNA expression levels by Real-time PCR (left) in KRT6A overexpressing and control H1299 cells. The G6PD mRNA expression level and G6PD promoter activity (right) in MYCN overexpressing and control H1299 cells. **(C)** Correlation of c-MYC with LSD1 (KDM1A), KRT6A and G6PD expression in NSCLC tumor tissues, calculated from http://gepia.cancer-pku.cn/.

The MYC family member c-MYC and MYCN are the most frequently deregulated oncogenes in human cancer and promote tumor progression through multiple levels of mechanisms in particular reprogrammed metabolism (Yoshida, 2020). Interestingly, we found that KRT6A overexpression augmented c-MYC/MYCN gene expression. Furthermore, c-MYC/MYCN significantly upregulated the expression of G6PD and enhanced its promoter activity, phenocopying the effect of KRT6A (Figures 6A,B). Next, correlation analysis indicated that expression of c-MYC and KRT6A, LSD1, G6PD were positively correlated in NSCLC tumor tissues (Figure 6C). These data indicated that KRT6A might induce G6PD expression by upregulating c-MYC/MYCN.

## Discussion

Recent studies revealed that high expression of KRT6A is associated with unfavorable prognosis of lung cancer patients (Xiao et al., 2017). A study by Yang et al. (2020) suggested that KRT6A promotes the growth and metastasis of lung adenocarcinoma through inducing the epithelial-mesenchymal transition. Here, our study has yielded similar results, by showing that KRT6A is upregulated in NSCLC tumors and promotes lung cancer cell proliferation and invasion *in vitro*. Consistently, KRT6A significantly decreases E-cadherin levels in lung cancer cells. Through clinical analysis, we found that the elevated expression of KRT6A can be used as a risk factor for predicting the survival outcome of NSCLC patients. Interestingly, another study confirmed our finding by showing that KRT6A inhibits the proliferation and invasion of lung adenocarcinoma cells, and high expression of KRT6A protein may be a prognostic marker for patients with lung adenocarcinoma (Xiao et al., 2020). Therefore, deeper investigations are warranted to explore the function of KRT6A in lung cancer progression.

Histone lysine-specific demethylase 1 (LSD1/KDM1A) has been considered as an important and promising anti-cancer target (Fang et al., 2019; Dai et al., 2020). LSD1 is overexpressed in various tumor types, and may induce cancer cell proliferation, invasion, and migration (Dai et al., 2020). Importantly, studies have shown that LSD1 is a potential therapeutic target for NSCLC. High levels of LSD1 are correlated with poor prognosis of NSCLC patients and the proliferation, migration, and invasion capabilities of tumor cells (Lv et al., 2012). Moreover, it was reported that LSD1+8a is a LSD1 isoform contributing to neural differentiation in small cell lung cancer (Jotatsu et al., 2017). In addition, Takagi et al. (2017) found that the LSD1 inhibitor T-3775440 inhibits the proliferation of lung cancer cells by disrupting the LSD1 interaction with the SNAG domain proteins INSM1 and GFI1B. Our previous work suggested that another LSD1 inhibitor ORY-1001 inhibits lung cancer cell growth and induces cell apoptosis by triggering HK2-mediated cellular events (Lu et al., 2018). In this study, our data revealed that LSD1 promotes KRT6A expression. Moreover, we found that ORY-1001 inhibits the invasion of NSCLC cells in a dose-dependent manner, which is reminiscent of a recent finding that ORY-1001 induces tumor regression in small cell lung cancer patient-derived xenograft model by regulating NOTCH (Augert et al., 2019).

The molecular mechanism whereby KRT6A expression promotes tumor progression is incompletely understood. Notably, studies have found that the pentose phosphate pathway (PPP) is an essential glucose metabolic pathway promoting tumor growth and metastasis (such as in lung cancer). G6PD as a crucial rate-limiting enzyme of PPP, greatly facilitates tumor invasion (Mele et al., 2018; Jin and Zhou, 2019). Nagashio et al. (2019) reported that the survival rate of lung adenocarcinoma patients with G6PD upregulation in the invasive frontier was significantly lower than those without G6PD upregulation. Multivariate analysis further showed that G6PD expression is an independent factor for patient prognosis (Nagashio et al., 2019). Our study revealed that overexpression of KRT6A could promote DNA synthesis, increase G6PD expression level, and enhance G6PD promoter activity. These results suggested that KRT6A may promote lung cancer progression by regulating G6PD.

It has been reported that c-MYC and MYCN are amplified and/or overexpressed in lung cancer. c-MYC and MYCN may be used as therapeutic targets for treating lung cancer patients (Masso-Valles et al., 2020). There are also research findings showing that c-MYC is involved in the regulation of G6PD expression (Yang et al., 2018). In this study, we observed that KRT6A could promote c-MYC/MYCN expression. Furthermore, c-MYC/MYCN significantly upregulates the expression of G6PD by stimulating its promoter activity. Correlation analysis indicated that expressions of c-MYC and KRT6A, LSD1, G6PD were all positively correlated in NSCLC tumors. Together, our results indicated that KRT6A might induce G6PD expression through the c-MYC/MYCN axis.

In summary, we discovered that KRT6A expression is frequently upregulated in lung cancer. KRT6A regulates the expression of G6PD through c-MYC/MYCN to promote the proliferation and invasion of lung cancer cells. The elevated expression of KRT6A can be used as a risk factor for predicting the clinical outcome of NSCLC patients. Our data provide additional evidences to support KRT6A and LSD1 as potential therapeutic targets for treating NSCLC.

## Data Availability Statement

The datasets presented in this study can be found in online repositories. The names of the repository/repositories and accession number(s) can be found in the article/Supplementary Material.

## Ethics Statement

The studies involving human participants were reviewed and approved by Ethics Committee of Wenzhou Medical University. The patients/participants provided their written informed consent to participate in this study.

## Author Contributions

All authors contributed significantly to this work, gave final approval of the version to be published and agreed to be accountable for all aspects of the work, participated in drafting the article or revising it critically for important intellectual content, agreed to submit the manuscript to the current journal, made substantial contributions to the conception and design of the study as well as the acquisition, analysis and interpretation of data.

## Conflict of Interest

The authors declare that the research was conducted in the absence of any commercial or financial relationships that could be construed as a potential conflict of interest.

## Footnotes

1 http://gepia.cancer-pku.cn/

2 http://kmplot.com/analysis
