# Supplementary figures and images for: KRT6A Promotes Lung Cancer Cell Growth and Invasion Through MYC-Regulated Pentose Phosphate Pathway
Source: Front Cell Dev Biol. 2021 Jun 21;9:694071. doi: 10.3389/fcell.2021.694071 (PMC8255478; doi:10.3389/fcell.2021.694071)

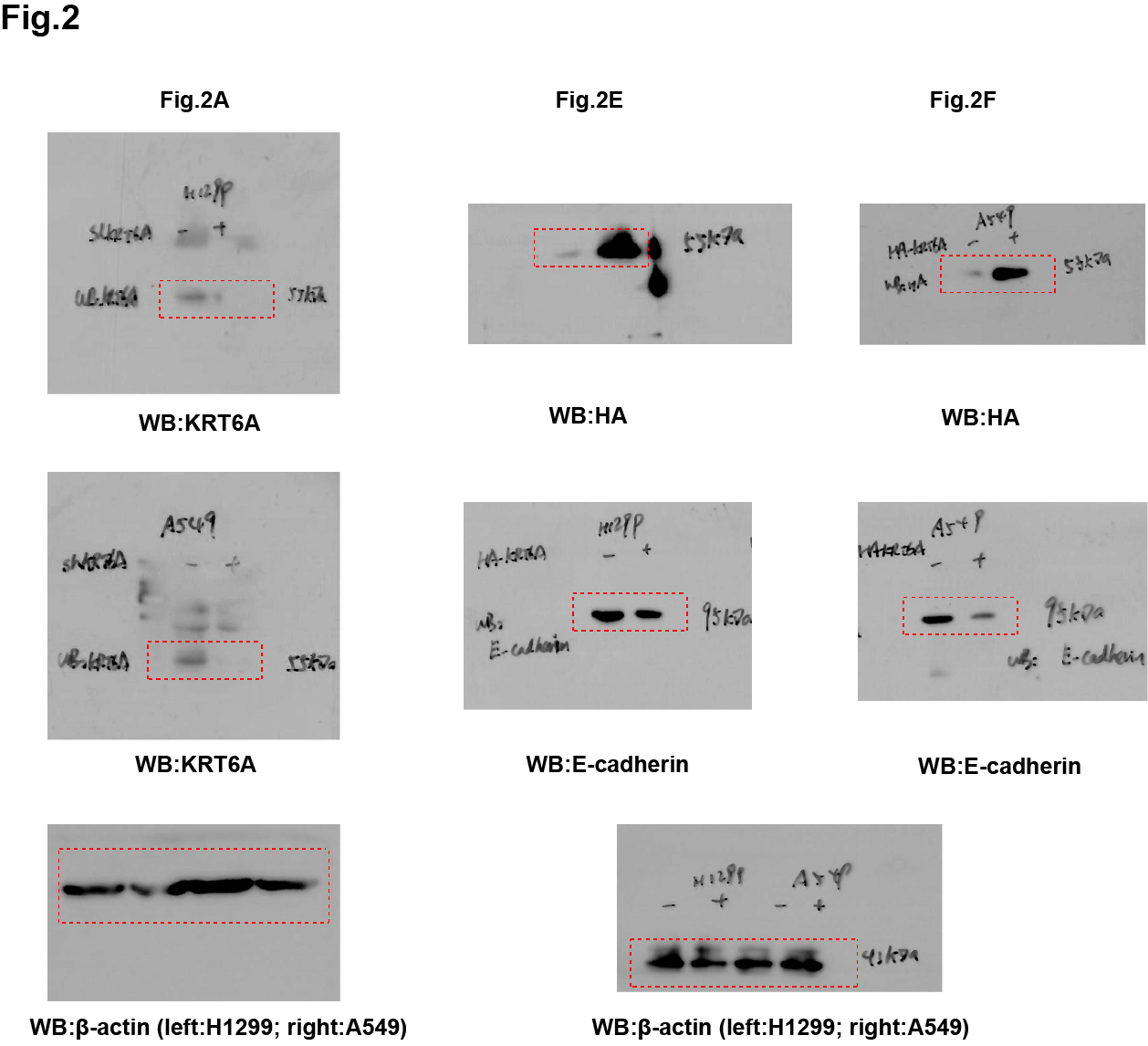

Supplement: Supplementary file 1 [file Image_1.TIF]

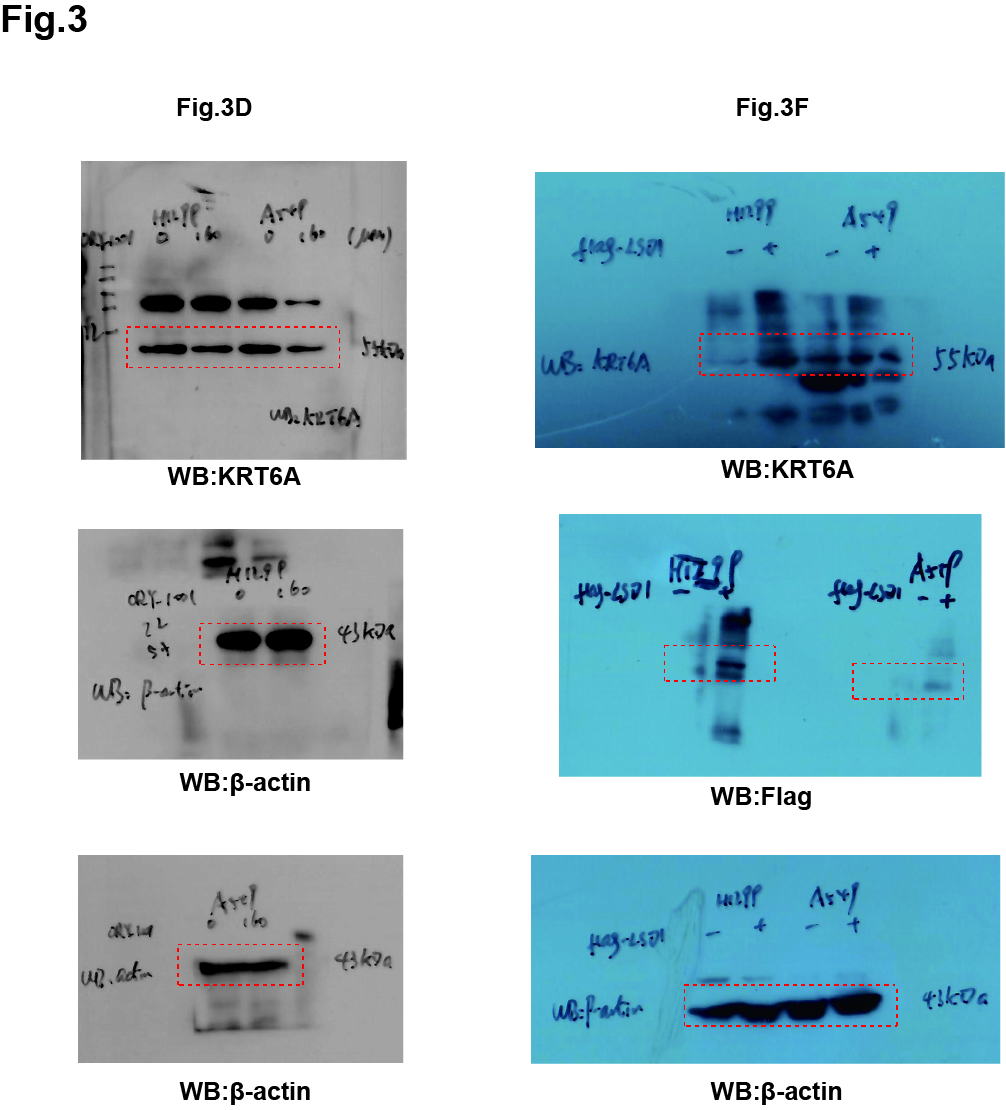

Supplement: Supplementary file 2 [file Image_2.TIF]

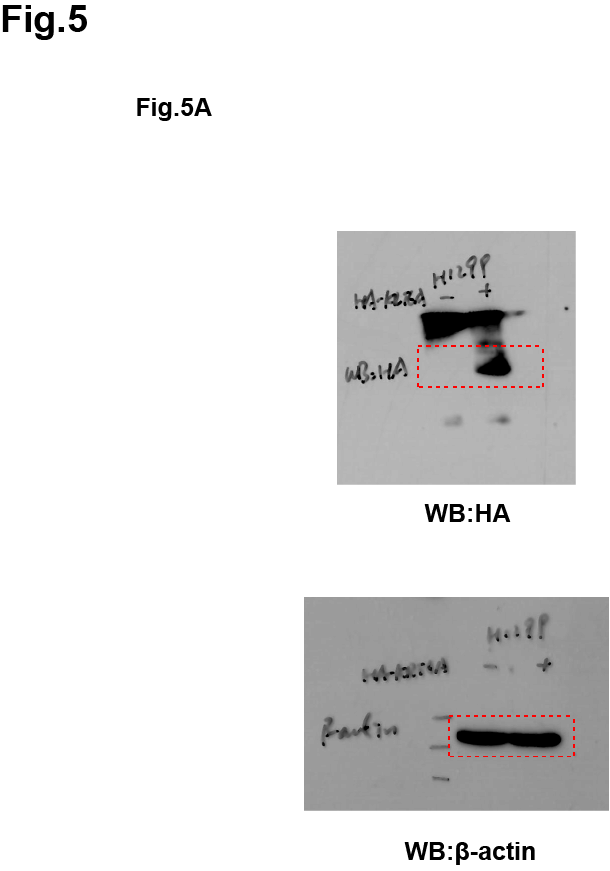

Supplement: Supplementary file 3 [file Image_3.TIF]
